# Pediatric Ileosigmoid Knotting: A Rare Culprit of Acute Abdominal Pain

**DOI:** 10.7759/cureus.42749

**Published:** 2023-07-31

**Authors:** Saleh A Ba-shammakh, Nimah A Rabai, Hasn M Haj-Freej, Waleed H Ghanem, Mohamad N Wahsheh

**Affiliations:** 1 Department of General Surgery, Princess Basma Teaching Hospital, Irbid, JOR; 2 Department of General Surgery, The Islamic Hospital, Amman, JOR; 3 Department of Pediatrics, Princess Rahma Hospital, Irbid, JOR

**Keywords:** colorectal anastomosis, surgical emergency, whirl signs, primary enteroenterostomy, ileosigmoid knotting

## Abstract

The present study focuses on ileosigmoid knotting (ISK), an infrequent but potentially lethal surgical emergency. ISK is more frequently observed in males and is prevalent in regions with high rates of sigmoid volvulus. The current medical scenario revolves around the intricate intertwining of the ileum and sigmoid colon, leading to a complicated volvulus. This condition often results in acute intestinal obstruction, which can subsequently cause bowel necrosis. The clinical presentation of the condition is characterized by symptoms such as abdominal distention, pain, vomiting, and obstipation, which can be misleading as they are similar to other common abdominal conditions. A delay in definitive diagnosis and intervention is likely, which can lead to severe consequences such as peritonitis, bowel necrosis, sepsis, and even septic shock. Our case report discusses an instance of ISK presenting as an acute abdomen requiring an emergency laparotomy and detorsion of the volvulus-knotted segment, followed by an elective sigmoidectomy. The significance of a heightened level of suspicion, prompt decision-making, and timely surgical intervention in achieving better patient outcomes cannot be overstated. The objective is to enhance the medical community's understanding of ISK, focusing on early diagnosis and effective treatment of this rare but life-threatening disease.

## Introduction

Ileosigmoid knotting (ISK), also known as compound or double volvulus, is a rare yet potentially lethal surgical emergency that results in a closed-loop intestinal obstruction, often involving the ileum and the sigmoid colon [1-2]. This condition arises when the ileum wraps around the base of the sigmoid colon, which can rapidly lead to gangrene in the affected gut segments [1,3]. ISK is primarily reported in Asian, Middle Eastern, and African nations. It poses a diagnostic challenge due to its clinical similarity to sigmoid volvulus and nonspecific radiographic findings [4-5]. Consequently, preoperative diagnoses are infrequent, with only 0-28% of cases correctly identified before surgery [1]. The average mortality rate for ISK is approximately 35.5%, which can increase in instances of gangrenous bowel or septic shock [1]. Factors potentially contributing to these mortality rates include delayed diagnoses, the urgency of required intervention, and occasionally, atypical presentations [1,3]. The cause of ISK remains a mystery, but several potential contributors are considered, such as a lengthy sigmoid colon with a slender stem, a small bowel mesentery allowing substantial mobility to the small intestine, and the intake of a high-fiber diet alongside an empty small bowel [6]. Late pregnancy, thick-banded Meckel diverticulitis, and ileocecal intussusception also contribute [5]. Through this report, our aim is to enhance understanding of this uncommon condition, encouraging earlier diagnosis and intervention. We emphasize the importance of distinguishing ISK from a simple sigmoid volvulus due to the contraindication of endoscopic decompression in ISK [2].

## Case presentation

A previously healthy nine-year-old male presented with acute abdominal pain for 24 hours, associated with abdominal distension and repetitive vomiting of approximately 30cc of bile. He had a history of chronic constipation; no blood or fecal material was detected in the vomitus. The patient was visibly distressed and exhibited signs of dehydration. On examination, the patient was alert, responsive, oriented, and appeared agitated. Vital signs revealed a pulse rate of 140 beats per minute, blood pressure of 90/60 mmHg, temperature of 38.5°C, and a respiratory rate of 25 breaths per minute. Oxygen saturation was measured at 99% on room air.

**Figure 1:**
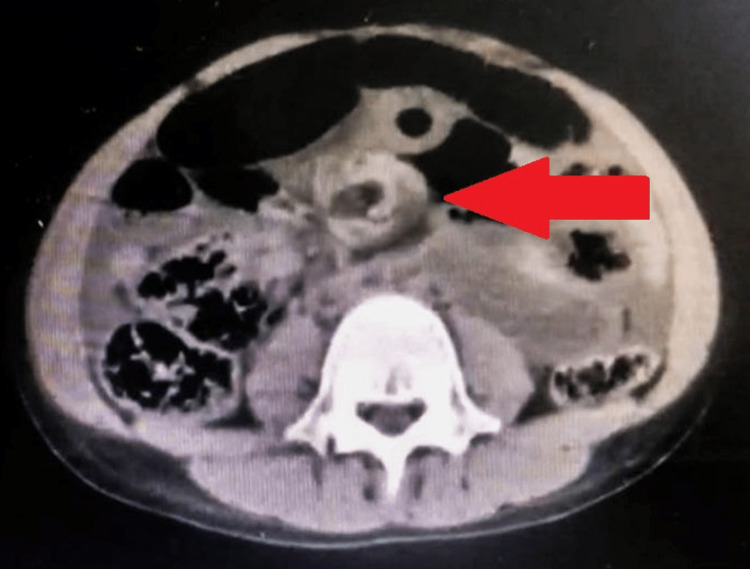
Abdominal CT scan Abdominal CT scan without contrast showing "whirl signs" at the level of the superior mesenteric artery, suggestive of midgut volvulus

Chest examination was clear, and abdominal examination revealed distention, rigidity, and guarding, with hypertympanic bowel sounds on auscultation, suggesting peritonitis. Per rectum examination showed good sphincter tone with normal stool on the tip of the finger. No blood was detected. Laboratory investigations showed a white blood cell (WBC) count of 18 x 10^9/L and a hemoglobin level of 13 g/dL. Kidney function tests were within normal range. Abdominal computed tomography (CT) scan from another hospital, performed without contrast, revealed "whirl signs" at the level of the superior mesenteric artery (Figure 1). This combination of clinical history and radiological findings confirmed a diagnosis of midgut volvulus.

**Figure 2:**
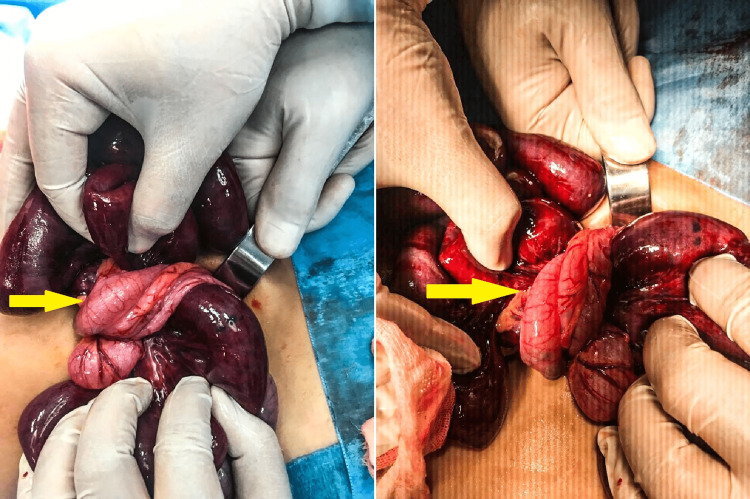
Intraoperative findings The sigmoid colon twisted anticlockwise around the Ileum

**Figure 3:**
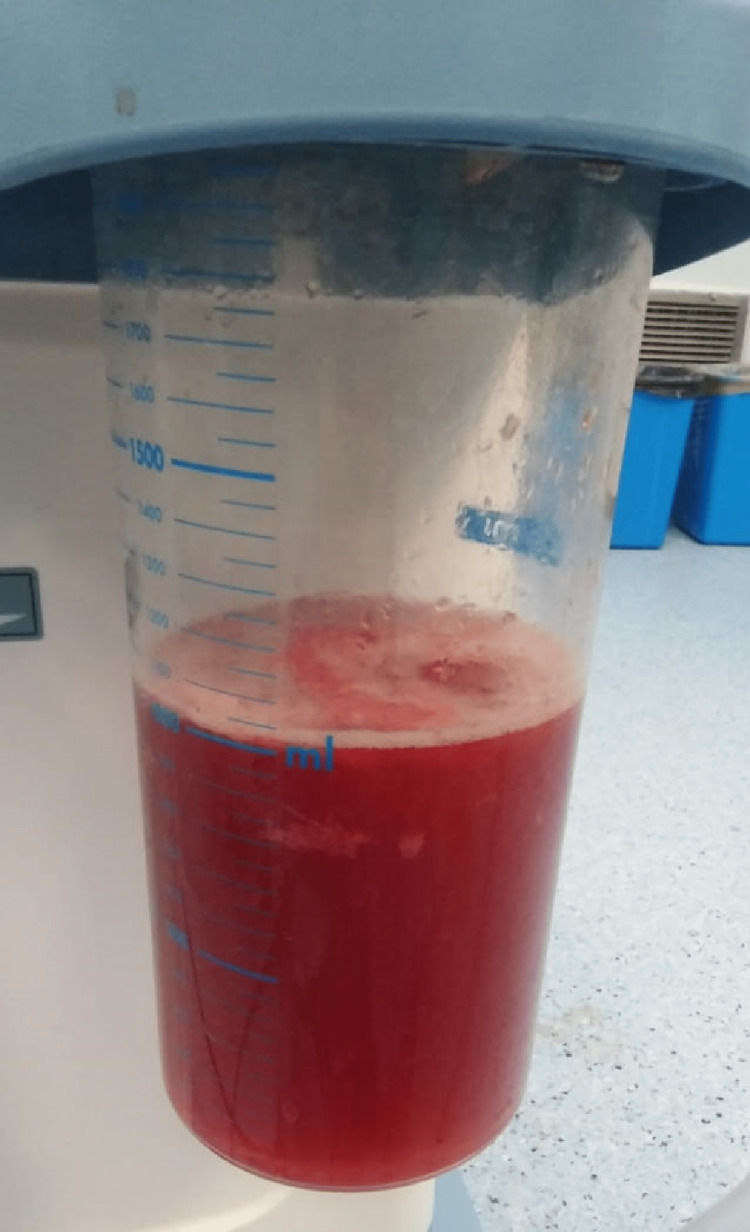
Suction bottle Collection of approximately 1000 ml of hemorrhagic fluid within the peritoneal cavity

**Figure 4:**
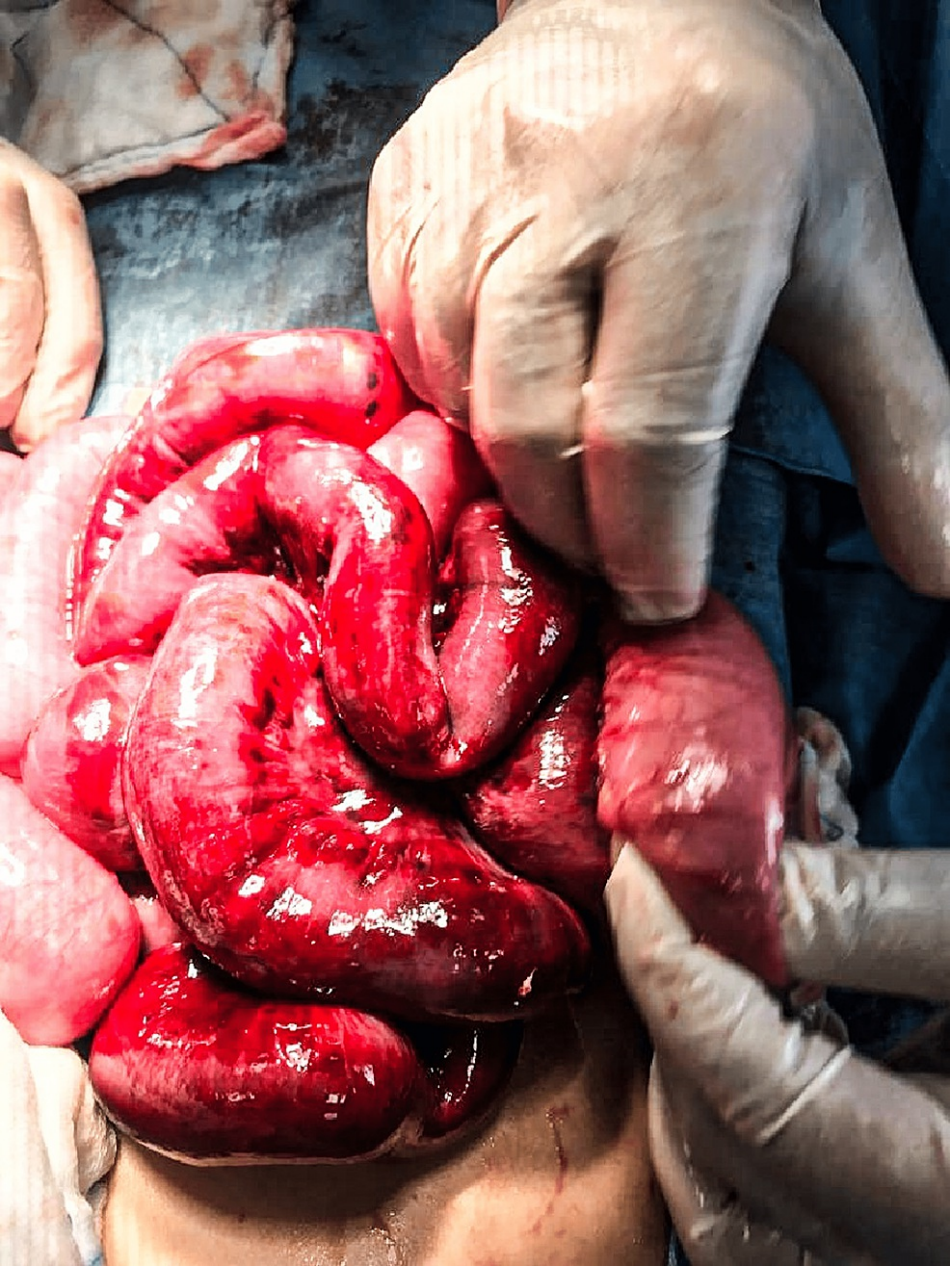
Post-detorsion Improved vascularity with no evidence of gangrenous changes

The patient was resuscitated with intravenous fluids upon transfer to our pediatric tertiary hospital. Broad-spectrum antibiotics were also administered. He underwent an exploratory laparotomy within an hour of admission. Intraoperatively, viable but ischemic ileal loops were noted twisted approximately 360 degrees anticlockwise around a normal, redundant sigmoid colon loaded with fecal material (Figure 2). Approximately 1000 ml of hemorrhagic fluid within the peritoneal cavity and pelvis was promptly suctioned (Figure 3). Post-detorsion, the twisted ileum demonstrated improved vascularity with no evidence of gangrenous changes (Figure 4). The patient was transferred to the surgical ward for close postoperative monitoring and was discharged seven days post surgery with no complications. His hemoglobin levels post-operation were within acceptable ranges of 11.5 g/dL and did not necessitate a blood transfusion.

An elective total sigmoidectomy with colorectal anastomosis was scheduled one month later. Despite significant intraoperative adhesions, a total sigmoidectomy was successfully performed, during which a 35 cm segment of the patient's sigmoid colon was removed, and a colorectal anastomosis was created. The patient was discharged on the fifth day after surgery, having experienced a smooth recovery. Follow-up appointments were regularly scheduled to keep a close watch on the patient's condition, during which no recurrence of the symptoms has been observed.

## Discussion

ISK, often known as a "double volvulus," is a rare but serious medical condition where the ileum and sigmoid colon intertwine and block the intestinal loop [1]. It can affect individuals from all demographics, including children [5]. ISK is categorized into four types (I - IV), based on the intestinal segments implicated in the knot formation. Type I, the most commonly seen, involves the ileum actively knotting around the static sigmoid colon. Conversely, type II has the sigmoid colon creating a knot around the ileum. Type III implicates both the ileocecal segment and the sigmoid colon in the knot, while in Type IV, the cause of the knotting is not well-defined [3]. A classification system was put forth by Atamanalp and his team in 2009, which considers risk and prognosis in terms of preoperative and operative elements. The first class includes patients with no risk factors, whereas the second class comprises patients with predisposing factors but without signs of shock or bowel gangrene. The third class includes patients exhibiting shock symptoms, and the fourth class includes those with bowel gangrene. The fifth and sixth classes involve patients with shock and gangrene of the ileum or sigmoid colon, respectively [3]. The patient was classified under type II and class II, with a history of chronic constipation [1].

ISK's clinical presentation typically encompasses abdominal pain, bloating, nausea, vomiting, and in severe instances, shock [4]. These symptoms, however, largely mirror other gastrointestinal disorders like sigmoid volvulus or small bowel obstruction, making ISK diagnosis complex before surgery. An emergency exploratory laparotomy is often the most conclusive diagnostic tool [1]. Although CT scans may reveal the characteristic whirl sign or medial deviation of the distal descending colon, more economical imaging options such as X-rays or ultrasound scans are frequently utilized, especially in regions with limited resources [3].

Prompt medical action is essential due to ISK's rapid progression to a gangrenous bowel. Initial measures include resuscitating the patient and stabilizing hemodynamics before surgical intervention [1]. The potential for bowel perforation or injury makes endoscopic reduction inappropriate [7]. Gangrenous bowel can complicate the unknotting procedure, increasing the risk of toxic bowel content leakage. Hence, the bowel should be clamped prior to dissection or resection [7]. The condition of the residual bowel then determines the surgical strategy. Options include primary colorectal anastomosis and primary enteroenterostomy, although, in certain situations, a diversionary ileostomy or colostomy might be necessary [8].

## Conclusions

ISK is a rare, potentially lethal surgical emergency demanding prompt diagnosis and decisive intervention. Our report stresses the need for a high clinical suspicion. A deeper understanding of ISK risk factors and early diagnostic strategies can improve patient outcomes in this complex and rapidly progressing condition.
